# Role of the high-affinity leukotriene B_4_ receptor signaling in fibrosis after unilateral ureteral obstruction in mice

**DOI:** 10.1371/journal.pone.0202842

**Published:** 2019-02-28

**Authors:** Mariko Kamata, Hideki Amano, Yoshiya Ito, Tomoe Fujita, Fumisato Otaka, Kanako Hosono, Kouju Kamata, Yasuo Takeuchi, Takehiko Yokomizo, Takao Shimizu, Masataka Majima

**Affiliations:** 1 Department of Pharmacology, Kitasato University School of Medicine, Sagamihara Kanagawa, Japan; 2 Department of Nephrology, Kitasato University School of Medicine, Sagamihara Kanagawa, Japan; 3 Department of Molecular Pharmacology, Kitasato University Graduate School of Medical Sciences, Kanagawa, Japan; 4 Department of Pharmacology and Toxicology, Dokkyo Medical University, Shimotsuga-gun, Tochigi, Japan; 5 Sagamiono Medical and Kidney Clinic, Sagamihara Kanagawa, Japan; 6 Department of Biochemistry, Juntendo University School of Medicine, Bunkyo-ku, Tokyo, Japan; 7 Department of Lipidomics, Faculty of Medicine, University of Tokyo, Bunkyo-ku, Tokyo, Japan; 8 Department of Lipid Signaling, National Center for Global Health and Medicine, Shinjuku-ku, Tokyo, Japan; Hopital Tenon, FRANCE

## Abstract

Leukotriene B_4_ (LTB_4_) is a lipid mediator that acts as a potent chemoattractant for inflammatory leukocytes. Kidney fibrosis is caused by migrating inflammatory cells and kidney-resident cells. Here, we examined the role of the high-affinity LTB_4_ receptor BLT1 during development of kidney fibrosis induced by unilateral ureteral obstruction (UUO) in wild-type (WT) mice and BLT1 knockout (BLT1^-/-^) mice. We found elevated expression of 5-lipoxygenase (5-LOX), which generates LTB_4_, in the renal tubules of UUO kidneys from WT mice and BLT1^-/-^ mice. Accumulation of immunoreactive type I collagen in WT UUO kidneys increased over time; however, the increase was less prominent in BLT1^-/-^ UUO kidneys. Accumulation of S100A4-positive fibroblasts increased temporally in WT UUO kidneys, but was again less pronounced in-BLT1^-/-^ UUO kidneys. The same was true of mRNA encoding transforming growth factor-β (TGF)-β and fibroblast growth factor (FGF)-2. Finally, accumulation of F4/80-positive macrophages, which secrete TGF-β, increased temporally in WT UUO and BLT1^-/-^ UUO kidneys, but to a lesser extent in the latter. Following LTB_4_ stimulation *in vitro*, macrophages showed increased expression of mRNA encoding TGF-β/FGF-2 and Col1a1, whereas L929 fibroblasts showed increased expression of mRNA encoding α smooth muscle actin (SMA). Bone marrow (BM) transplantation studies revealed that the area positive for type I collagen was significantly smaller in BLT1^-/—^BM→WT than in WT-BM→WT. Thus, LTB_4_-BLT1 signaling plays a critical role in fibrosis in UUO kidneys by increasing accumulation of macrophages and fibroblasts. Therefore, blocking BLT1 may prevent renal fibrosis.

## Introduction

Unilateral ureteral obstruction (UUO) is an experimental animal model of renal fibrosis that mimics the pathogenesis of chronic obstructive nephropathy in humans. The hydrostatic pressure resulting from the obstruction triggers expression of chemokines in tubular epithelial cells [1], followed by increased interstitial capillary permeability [2], infiltration by interstitial inflammatory cells [3], myofibroblast activation, and extracellular matrix deposition [4–6]. Progressive fibrosis, loss of renal parenchyma due to capillary rarefaction [4], and tubular cell death via apoptosis and necrosis [7] also occur. Despite these severe changes in the obstructed kidney, the animal remains healthy because the contralateral kidney is fully functional. Indeed, unlike renal ablation models [8], UUO model mice do not have uremia. Therefore, the UUO model is ideal for studying the histopathological and molecular changes underlying tubulointerstitial damage, a process that closely resembles deterioration of renal function in humans with chronic kidney disease [9–11].

Macrophages are rarely present in the healthy renal cortex [12, 13]. However, within hours of ureteral obstruction, a large number of blood-derived macrophages accumulate in the tubulointerstitial space [14]. This cellular infiltration is preceded by local expression of chemokines [1, 15], chemokine receptors [1], and adhesion molecules [16, 17]. Despite the accumulation of strong correlative data, there are few functional studies describing the role of these infiltrating macrophages and lipid mediators in UUO-induced fibrosis. A previous report showed that prostaglandin (PG) E_2_, a major metabolite of arachidonic acid, suppresses tubulointerstitial fibrosis via EP4 [18]. Furthermore, EP4 signaling suppressed accumulation of macrophages in the kidneys following induction of UUO. A recent report suggests that bone marrow (BM)-derived macrophages, which expressed c phospholipase (PLA)_2_α, upstream of the 5-lipoxygenase (5-LOX) pathway, exacerbate fibrosis in the UUO kidney [19].

Leukotrienes (LTs) are metabolites of arachidonic acid that are generated via the 5-LOX (EC 1.13.11.34, 5-LOX) pathway. LTB_4_ is a well-characterized and potent chemoattractant for leukocytes, particularly neutrophils and monocytes [20]; as such, it plays a pivotal role in the pathogenesis of inflammatory and immune diseases such as asthma [21], sepsis [22], and atherosclerosis [23, 24]. Previously, we showed that LTB_4_ is a potent inducer of neutrophil extravasation into the interstitial space in certain *in vivo* models [25–28]. LTB_4_ exerts its biological activity through two distinct receptors: LTB_4_ receptor type-1 (BLT1), a high-affinity LTB_4_ receptor highly expressed in leukocytes, and BLT2, a low-affinity LTB_4_ receptor expressed more ubiquitously than BLT1 in human tissues [29–31].

　Hemodynamic changes, which were dependent on the 5-LOX pathway, were described in a rat model of bilateral ureteral obstruction [32]. Although, blocking LTB_4_ activity reduced fibrosis in bleomycin-treated lungs [33], the role of BLT1 signaling in UUO-induced fibrosis remains unclear.

Here, we examined the role(s) of BLT1 signaling in development of fibrosis in a BLT1 knockout (BLT1^-/-^) mouse model of UUO [34]. We noted significantly less accumulation of type I collagen in kidneys of BLT1^-/-^ mice with UUO than in those of wild-type (WT) mice. We concluded that LTB_4_-BLT1 signaling plays a role in tubulointerstitial fibrosis of the kidney, possibly via upregulation of TGF-β and increased recruitment of myofibroblasts and fibroblasts. Thus, blocking BLT1 signaling may prevent fibrosis in those with chronic kidney disease.

## Materials and methods

### Animals and surgery

BLT1^-/-^ mice were developed as described previously [34]. Male C57BL/6 WT mice and BLT1^-/-^ mice (8 weeks old) were used. UUO surgery was performed under inhalation anesthesia of isoflurane mixed with air and its adequacy was monitored from the disappearance of the pedal withdrawal response. A median abdominal incision was made, and the left proximal ureter was ligated at two points using 3–0 silk. The incision was closed with wound clips (AUTOCLIP, 9 mm; ALZET, Cupertino, CA, USA). Sham-operated mice had the ureter exposed but not ligated [35]. All experimental procedures were approved by the Animal Experimentation and Ethics Committee of the Kitasato University School of Medicine (2018–166), and were performed in accordance with the guidelines for animal experiments established by the Kitasato University School of Medicine and conformed to the Guide for the Care and Use of Laboratory Animals published by the US National Institutes of Health (NIH Publication No. 85–23, revised 1996). The mice were maintained at constant humidity (60 ± 5%) and temperature (22°C ± 1) on a 12-h light/ dark cycle. All animals were provided with food and water ad libitum. The total number of mice used in this experiment is 166. The number of mice per group is from 4 to 20. At the end point of the experiments, mice were sacrificed under inhalation anesthesia of isoflurane mixed with air. Mice exhibiting symptoms of infection including suppressed appetite, purulent discharge from the wound were removed from the study prior to the study endpoint.

### Tissue harvesting

Kidney samples were collected on Days 0, 1, 3, 5, 7, 10, and 14 after UUO. Day 0 kidney samples were collected without the need for surgical procedures. All mice were anesthetized with isoflurane and perfused with PBS via the left ventricle. The left kidney was harvested immediately and cut into transverse sections for RT-PCR, paraffin embedding, freezing, and Sircol collagen assays.

### Histological examination

Kidney tissues were fixed overnight at 4°C in 4% paraformaldehyde and embedded in paraffin. Paraffin-embedded tissues were cut into 4 μm sections and stained with H&E and Sirius red. Kidney cortex thickness was measured by investigators blinded using Image J software.

### Immunofluorescence staining

Unfixed kidney tissues were frozen immediately in liquid nitrogen. Samples were cut into 4 μm sections from the cortical side, blocked with 1% BSA in PBST (0.1% Triton X-100 in PBS) for 1 h at room temperature, and incubated overnight at 4°C with an anti-type I collagen antibody (1:100 dilution; Abcam, Cambridge, UK; ab21286). After washing in PBS, the sections were incubated for 1 h at room temperature with Alexa Fluor 488-Donkey anti-rabbit IgG (1:500 dilution; Molecular Probes, Eugene, OR, USA). Five randomly selected cortical interstitial fields from each animal were photographed (at ×400 magnification), excluding the glomeruli and large vessels. The immunoreactive interstitial area was calculated using Image J software and expressed as a percentage of the total area. Periodate-lysine-paraformaldehyde tissues were fixed for 2 h at 4°C, frozen in liquid nitrogen, cut into 10 μm sections, and stained as described above with one of the following primary antibodies: anti-5-LOX (1:100 dilution; Novus Biologicals, Littleton, CO, USA; NB 100–92138), anti-CXCL12 (1:100 dilution; eBioscience, San Diego, CA, USA; 14–7992), anti-F4/80 (1:200 dilution; Santa Cruz Biotechnology, Inc., Dallas, TX, USA; sc-52664) or anti-Gr1 IgG2b (1:100 dilution AbD Serotec, Raleigh, NC, USA;RB6-8C5). After washing in PBS, the sections were incubated for 1 h at room temperature with one of the following secondary antibodies: Alexa Fluor 488-Donkey anti-Rabbit IgG (1:500 dilution, Molecular Probes) or Alexa Fluor 568-Donkey anti-Rat IgG (1:500 dilution, Molecular Probes).

### Immunohistochemistry

Paraffin-embedded tissues were cut into 4 μm sections, deparaffinized, and rehydrated. Endogenous peroxidase was quenched by immersion for 30 min in a 1% solution of hydrogen peroxide in methanol. After washing in ion-exchanged water, antigen retrieval was performed by microwaving three times for 5 min in citrate buffer solution (pH 6.0). The sections were then incubated for 10 min with Protein Block, Serum-Free (DAKO, Glostrup, Denmark), followed by an overnight incubation at 4°C with an anti-S100A4 antibody (1:400 dilution; Abcam, ab27957), anti-F4/80 (1:200 dilution; Santa Cruz Biotechnology, Inc., Dallas, TX, USA; sc-52664) or anti-Gr1 IgG2b (1:100 dilution AbD Serotec, Raleigh, NC, USA;RB6-8C5). After washing in PBS, the sections were incubated for 30 min at room temperature with N-Histofine Simple Stain MAX PO (R) (Nichirei Biosciences, Inc., Tokyo, Japan). Immune complexes were then detected with 3, 3’-diaminobenzidine tetrahydrochloride (DAB), and sections were counterstained with methyl green. The number of S100A4-positive interstitial cells in five random cortical fields (×200 magnification) per sample was counted. All images were captured by Biozero BZ-9000 series microscope (Keyence, Tokyo, Japan).

### Sircol collagen assay

The total amount of soluble collagen was measured using a Sircol collagen assay kit (Biocolor, Antrim, UK). Kidney samples were frozen immediately with in liquid nitrogen immediately and stored at -80°C until use. All measurements were performed in duplicate and results were expressed as μg of collagen/mg of kidney cortex.

### Real-time RT-PCR

Total RNA was extracted from decapsulated kidney tissues using TRIzol reagent (Gibco-BRL; Life Technologies, Rockville, MD, USA), and single-stranded cDNA was generated from 1 μg of total RNA via reverse transcription using the ReverTra Ace qPCR RT Kit (TOYOBO CO., LTD., Osaka, Japan), according to the manufacturer’s instructions. Real-time PCR was performed using SYBR Premix Ex Taq II (Tli RNaseH Plus; Takara Bio, Inc., Shiga, Japan). The gene-specific sequences are described in Table 1. Expression of target genes was normalized to that of GAPDH.

**Table 1:** Primers used for reverse transcription and quantitative PCR.

Mouse	Forward primer sequence	Reverse primer sequence
gene	5’-3’	5’-3’
GAPDH	ACATCAAGAAGGTGGTGAAGC	AAGGTGGAAGAGTGGGAGTTG
Col1a1	AGGCATAAAGGGTCATCGTG	GACCGTTGAGTCCGTCTTTG
Col3a1	AGGCAACAGTGGTTCTCCTG	GACCTCGTGCTCCAGTTAGC
5-LOX	TCATTGAGAAGCCAGTGAAGG	GTTGGGAATCCTGTCTGGTGA
BLT1	GGCTGCAAACACTACATCTCC	TCAGGATGCTCCACACTACAA
CD3	ACTGGAGCAAGAATAGGAAGG	ATAGTCTGGGTTGGGAACAGG
F4/80	TATCTTTTCCTCGCCTGCTTC	CACCACCTTCAGGTTTCTCAC
S100A4	TGGGGAAAAGGACAGATGAAG	ATGCAGGACAGGAAGACACAG
αSMA	GAAGAGCTACGAACTGCCTGA	TGAAAGATGGCTGGAAGAGAG
CXCL12	GCATCAGTGACGGTAAACCAG	GCACAGTTTGGAGTGTTGAGG
CXCR4	CTCTGAAGAAGTGGGGTCTGG	AAGTAGATGGTGGGCAGGAAG
TGF-β	AACAATTCCTGGCGTTACCTT	TGTATTCCGTCTCCTTGGTTC
FGF-2	GGCTGCTGGCTTCTAAGTGTG	TTCCGTGACCGGTAAGTATTG

### Serum levels of blood urea nitrogen and creatinine

Blood was drawn from the heart and then centrifuged and stored at −20°C until use. Levels of serum blood urea nitrogen (BUN) and creatinine (Cre) were measured using a Dri-Chem 7000 Chemistry Analyzer System (Fujifilm, Tokyo, Japan).

### Collection of peritoneal macrophages

Thioglycolate-induced peritoneal macrophages were collected from 8–12-week-old WT mice. In brief, 2 ml of 4% thioglycolate medium was injected into the peritoneal cavity. After 3 days, the peritoneal cavity was washed three times with 5 ml of PBS. Cells in the lavage fluid were washed and suspended in RPMI 1640 medium containing 10% FCS and then placed in 12-well culture plates (1 × 10^6^ cells/well). After incubation at 37°C in 5% humidified CO_2_ for 16 h, the plates were washed with PBS to remove non-adherent cells. Approximately 60% of cells remained adherent and were used for subsequent experiments.

### Cell culture and treatments

At 16 h after plating, cells were washed twice with PBS and incubated for 2 h in serum-free medium. Cells were then stimulated for 12 h with LTB_4_ (0.1, 1, or 10 nM) or serum-free medium (control). Total mRNA was isolated from cells using TRIzol reagent, and mRNA expression was measured by real-time RT-PCR. Thioglycolate-induced peritoneal macrophages (after removal of non-adherent cells) were stimulated with LTB_4_, and expression of TGF-β, FGF-2, αSMA, and Col1a1 mRNA was measured. Murine fibroblasts (L929) were purchased from the Cell Bank at RIKEN BioResource Center (Ibaraki, Japan). Cells were suspended in DMEM containing 10% FCS, plated in 6-well culture plates (3 × 10^5^ cells/well), and stimulated with LTB_4_ and TGF-β, and expression of TGF-β, FGF-2, αSMA, and Col1a1 mRNA was measured.

### BM transplantation

BM transplantation experiments were carried out as described previously [36]. In brief, donor BM was obtained by flushing the femoral and tibial cavities of WT mice and BLT1^-/-^ mice with PBS. The flushed BM cells were dispersed and resuspended in PBS at a density of 1 × 10^6^ cells/100 μl. Both WT and BLT1^-/-^ mice were lethally irradiated with 9.5 Gy X-rays using an MBR-1505 R X-ray irradiator (Hitachi Medico, Tokyo, Japan) equipped with a filter (copper, 0.5 mm; aluminum, 2 mm). The cumulative radiation dose was monitored. BM mononuclear cells from WT and BLT1^-/-^ mice (2 × 10^6^ cells/200 μl) were transplanted into irradiated WT and BLT1^-/-^ mice via the tail vein.

### Statistical analysis

All results are expressed as the mean ± SEM. Comparisons between two groups were performed using Student’s *t* test. Comparisons between multiple groups were performed using one-way ANOVA, followed by Tukey’s *post-hoc* test. P values <0.05 were considered statistically significant.

## Results

### Development of fibrosis in WT mouse kidneys after UUO

**Fig 1:**
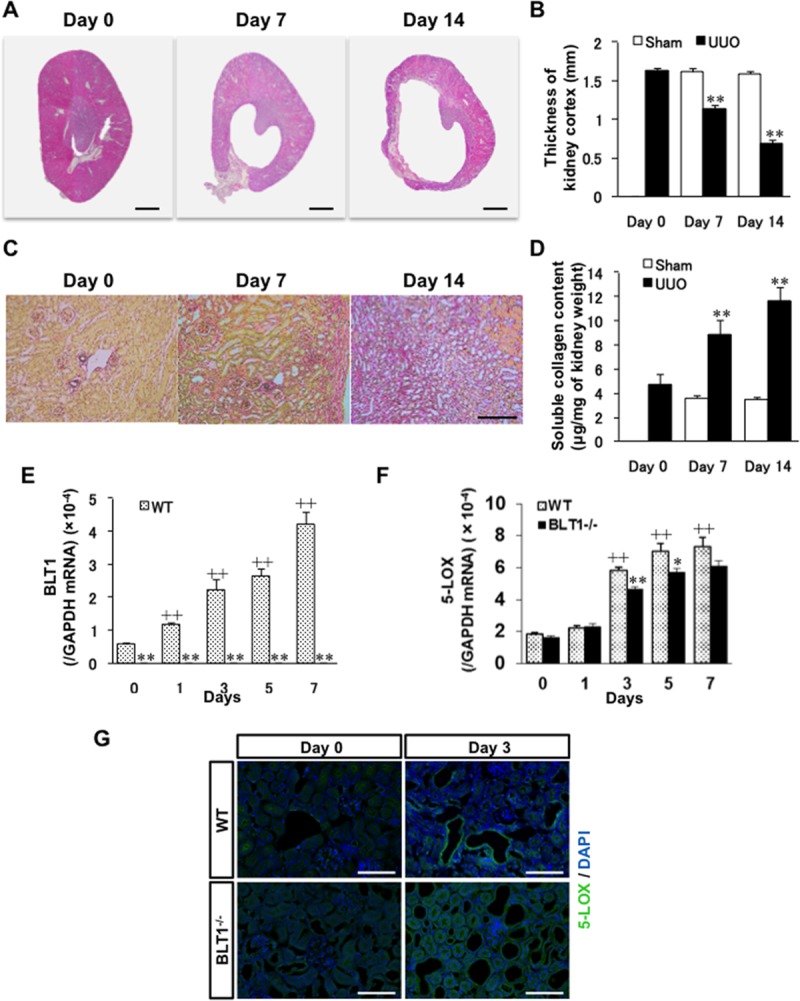
Effect of BLT1 and 5-LOX on development of fibrosis after UUO. (A) Photomicrographs showing H&E-stained transverse sections of WT kidney. Scale bars = 500 μm. (B) Changes in thickness of the WT UUO kidney cortex. Data are expressed as the mean ± SEM (n = 4 mice/group). **P<0.01, *vs*. sham. (C) Typical images showing Sirius red staining of WT kidney cortex after UUO. Scale bars = 200 μm. (D) Soluble collagen in the WT UUO kidney cortex measured in the Sircol collagen assay. Data are expressed as the mean ± SEM (n = 6 mice/group). **P<0.01, *vs*. Day 0. (E, F) Expression of mRNA encoding BLT1 (E) and 5-LOX (F) in UUO kidneys, as measured by real-time RT-PCR. Data are expressed as the mean ± SEM (WT; Day 0: n = 8, Day 1, 3, 5: n = 12, Day7: n = 12 BLT1; Day 0: n = 8, Day 1, 3, 5: n = 12, Day 7: n = 10 mice per group). *P<0.05 and **P<0.01, *vs*. WT; ^+^P<0.05 and ^++^P<0.01, *vs*. Day 0 WT. (G) Images of sections of WT and BLT1^-/-^ UUO kidney cortex immunostained with an anti-5-LOX antibody on Days 0 and 3. Epithelial cells in dilated renal tubules and some interstitial cells stained positive for 5-LOX. Scale bars = 200 μm.

Following induction of UUO, the thickness of the kidney cortex in WT mice decreased gradually (Fig 1A) and was significantly smaller than that in sham-operated mice at Days 7 and 14 (Day 7: 1.14±0.05 vs.1.62±0.04 mm, P<0.0001, Day 14: 0.68±0.05 vs.1.59±0.03 mm, P<0.0001, Fig 1B). Sirius red staining demonstrated that areas of collagen deposition around dilated renal tubules increased in a temporal manner (Fig 1C), and the Sircol collagen assay showed that collagen levels on Days 7 and 14 were significantly higher than sham-operated mice (Day 7: 8.85±1.20 vs.3.61±0.55μg/mg of kidney weight, P = 0.018, Day 14: 11.62±1.15 vs.3.47±0.16μg/mg, P = 0.0007, Fig 1D).

### Expression of BLT1 and 5-LOX increases in UUO kidneys

To study the role of LTB_4_-BLT1 signaling in the UUO kidney, we examined expression of 5-LOX (an enzyme upstream of LTB_4_) and BLT1. Expression of BLT1 mRNA in WT UUO kidneys on Day 1 was markedly higher than that on Day 0 (P<0.0001), while BLT1 mRNA levels in BLT1^-/-^ UUO kidneys were negligible throughout the experimental period (Fig 1E). By contrast, expression of 5-LOX increased in both WT and BLT1^-/-^ mice from Day 3, although levels in BLT1^-/-^ mice were significantly lower than those in WT mice at Days 3 and 5 (Day 3: P = 0.0002, Day5: P = 0.03, Fig 1F). Immunostaining of WT UUO kidneys and BLT1^-/-^ UUO kidneys for 5-LOX at Day 3 revealed that dilated tubule epithelial cells in WT mice were positive, as were some interstitial cells. By contrast, there were fewer 5-LOX-positive cells in dilated tubules and tubulointerstitial areas of BLT1^-/-^ mice (Fig 1G).

### Tubulointerstitial fibrosis in BLT1^-/-^ mice is reduced after UUO

**Fig 2:**
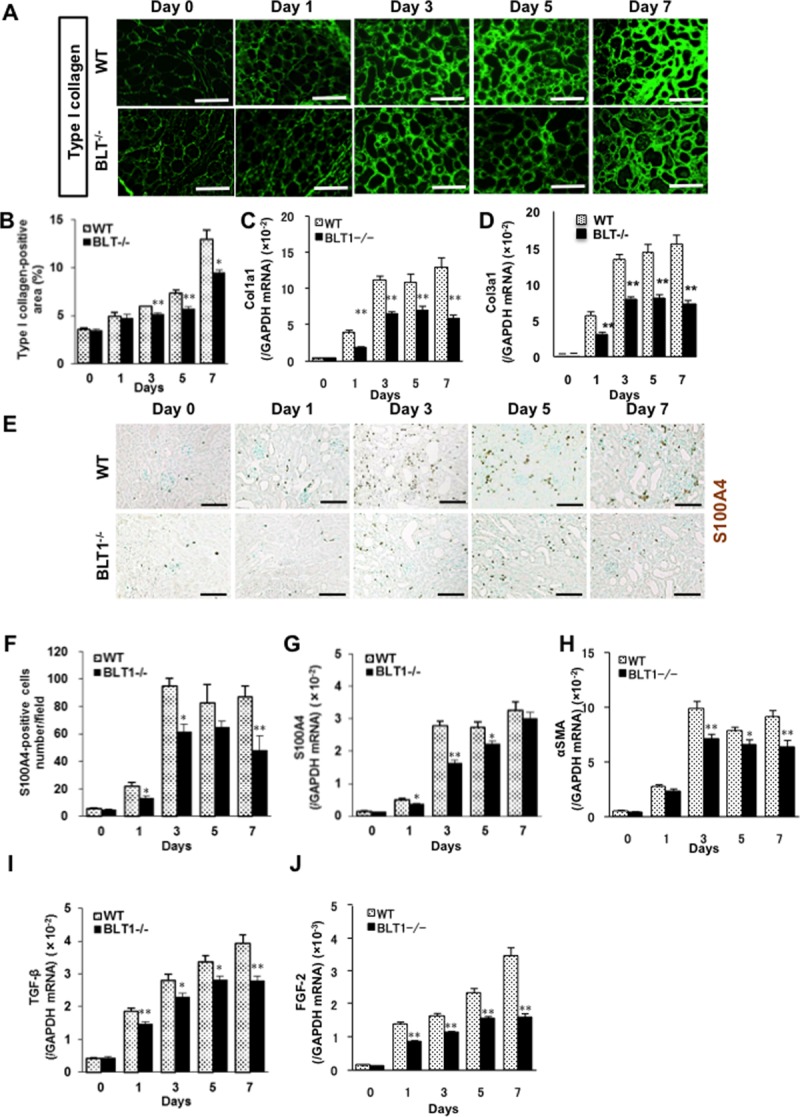
Tubulointerstitial fibrosis is less severe in BLT1^-/-^ mice following UUO. (A) Representative images of kidney cortex from WT and BLT1^-/-^ mice immunostained with an anti-type I collagen antibody. Scale bars = 50 μm. (B) Temporal changes in the area of immunoreactive collagen within tubulointerstitial spaces (expressed as % total area, excluding glomeruli and large vessels). Data are expressed as the mean ± SEM (n = 4 mice/group). *P<0.05 and **P<0.01, *vs*. WT. Expression of Col1a1 mRNA (C) and Col3a1 (D) mRNA in WT and BLT1^-/-^ mice kidneys, as measured by real-time PCR. Data are expressed as the mean ± SEM (WT; Day 0: n = 8, Day 1, 3, 5, 7: n = 16 BLT1; Day 0: n = 8, Day 1, 3, 5: n = 16, Day 7: n = 14 mice per group) *P<0.05 and **P<0.01, *vs*. WT. (D) Representative images showing S100A4 staining in WT and BLT1^-/-^ kidneys after UUO. Scale bars = 100 μm. (E) Changes in the number of S100A4-positive cells in WT UUO and BLT1^-/-^ UUO kidneys. (F) The number of S100A4-positive cells was significantly lower in BLT1^-/-^ UUO kidneys. Data are expressed as the mean ± SEM (n = 4 mice/group). (G, H) Expression of mRNA encoding S100A4 (G) and αSMA (H) in UUO kidneys, as measured by real-time PCR. Data are expressed as the mean ± SEM (WT; Day 0: n = 7, Day 1: n = 12, Day 3, 5, 7: n = 18 BLT1; Day 0: n = 7, Day 1: n = 12, Day 3, 5: n = 16, Day 7: n = 18 mice per group). *P<0.05 and **P<0.01, *vs*. WT. H, I) Expression of mRNA encoding TGF-β (I) and FGF-2 (J) in mouse kidneys after induction of UUO. Data are expressed as the mean ± SEM (TGF-β: WT; Day 0: n = 8, Day 1: n = 16, Day 3, 5, 7: n = 20 BLT1; Day 0: n = 8, Day 1: n = 14, Day 3, 5: n = 20, Day7: n = 18 mice per group. FGF-2: WT; Day 0: n = 8, Day 1, 3, 5, 7: n = 16 BLT1; Day 0: n = 8, Day 1, 3, 5, 7: n = 16 mice per group). *P<0.05 and **P<0.01, *vs*. WT mice.

To examine the role of BLT1 signaling in collagen accumulation, we examined kidney fibrosis in WT and BLT1^-/-^ mice after UUO. Immunostaining of type I collagen increased in WT kidneys after induction of UUO (Fig 2A). Quantitative analysis of the immunoreactive renal interstitial area revealed an increase in the percentage positive area after induction of UUO in both WT and BLT^-/-^ mice; however, the area of type I collagen was significantly lower in BLT1^-/-^ mice than in WT mice from Day 3 (Day 3: 5.95±0.05 vs.5.13±0.11%, P<0.001, Day 5: 7.36±0.30 vs.5.66±0.10, P = 0.004, Day 7: 12.9±0.99 vs.9.46±0.29, P = 0.02, WT vs. BLT1, respectively; Fig 2B). Furthermore, expression of mRNA encoding Col1a1 in WT UUO and BLT^-/-^ UUO kidneys increased in a temporal manner, but was significantly lower in BLT1^-/-^ mice from Day 1 (Day 1: <0.0001, Day 3: P<0.0001, Day 5: P = 0.008, Day 7: P = 0.0002, WT vs. BLT1, respectively; Fig 2C). Moreover, expression of mRNA encoding Col3a1 in WT UUO kidneys significantly increased as compared to BLT1^-/-^ UUO kidneys (Fig 2D)

### BLT1-dependent accumulation of fibroblasts in UUO kidneys

S100A4-positive fibroblasts accumulated in the interstitial tissues of WT UUO and BLT1^-/-^ UUO kidneys (Fig 2E). The number of S100A4-positive cells in immunohistochemical specimens from WT UUO kidneys increased in a time-dependent manner; however, there were significantly fewer S100A4-positive cells in BLT1^-/-^ UUO kidneys than in WT UUO kidneys at Days 1, 3, and 7 (Day 1: 21.9±2.9 vs.13.0±1.8 cells/field, P = 0.04, Day 3: 94.7±5.7 vs.61.8±5.4, P = 0.006, Day 7: 87.1±7.9 vs.48.1±10.8, P = 0.03, WT vs. BLT1, respectively; Fig 2F). In addition, expression of S100A4 mRNA in WT UUO kidneys increased, whereas that in BLT1^-/-^ UUO kidneys was suppressed, at Days 1, 3, and 5 (Day 1: P = 0.017, Day 3: P<0.0001, Day 5: P = 0.02, WT vs. BLT1^-/-^, respectively; Fig 2G). Changes in S100A4 mRNA levels mirrored changes observed upon immunohistochemical analysis. Expression of mRNA encoding αSMA, a myofibroblast marker, increased in WT UUO kidneys; however, this increase was significantly lower in BLT1^-/-^ UUO than in WT UUO kidneys after Day 3 (Day 3: P = 0.008, Day 5: P = 0.012, Day 7: P = 0.003, WT vs. BLT1^-/-^, respectively; Fig 2H).

### Upregulation of TGF-β and FGF-2 in UUO kidneys is BLT1-dependent

Several growth factors are expressed in UUO kidneys [10, 37]. Real-time PCR showed that expression of TGF-β mRNA in WT UUO kidneys increased from Day 1 post-induction, whereas the increases in BLT1^-/-^ mice were significantly lower (Day 1: P = 0.006, Day 3: P = 0.03, Day 5: P = 0.02, Day 7: P = 0.002, WT vs. BLT1^-/-^, respectively; Fig 2I). The same was true for FGF-2 mRNA (Day 1, 3, 5, 7: P<0.001, WT vs. BLT1^-/-^, respectively; Fig 2J).

### Accumulation of macrophages in UUO kidneys is BLT1-dependent

**Fig 3:**
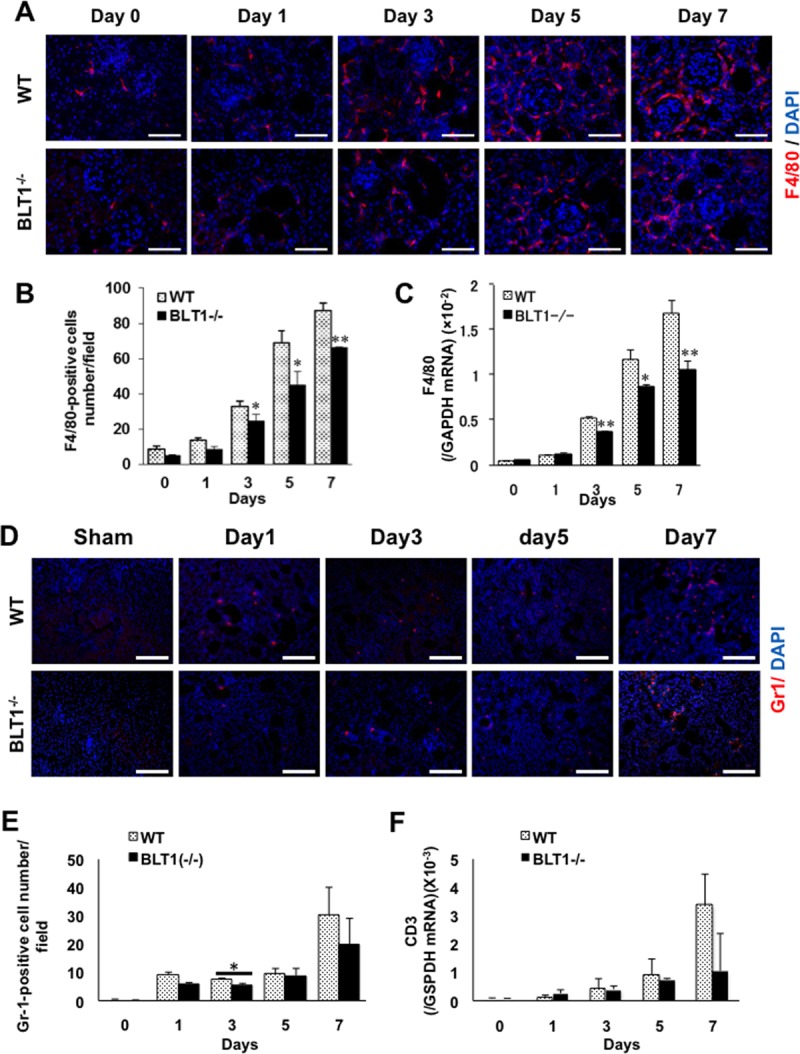
BLT1-induced accumulation of macrophages in UUO kidneys. (A) Representative images showing F4/80 staining of WT UUO and BLT1^-/-^ UUO kidneys. Scale bars = 50 μm. (B) Changes in the number of F4/80-positive cells in WT and BLT1^-/-^ kidneys after UUO treatment. Data are expressed as the mean ± SEM (n = 4 mice/group). (C) Expression of mRNA encoding F4/80 in UUO kidneys from WT and BLT1^-/-^ mice. Data are expressed as the mean ± SEM (WT; Day 0: n = 8, Day 1: n = 10, Day 3: n = 16, Day 5: n = 12, Day 7: n = 16 BLT1; Day 0: n = 8, Day 1: n = 12, Day 3: n = 16, Day 5: n = 11, Day7: n = 14 mice per group). *P<0.05 and **P<0.01, *vs*. WT mice. (D) Representative images showing Gr1 staining of WT UUO and BLT1^-/-^ UUO kidneys. Scale bars = 50 μm. (E) Changes in the number of Gr1-positive cells in WT and BLT1^-/-^ kidneys after UUO treatment. Data are expressed as the mean ± SEM (n = 4 mice/group). (F) Expression of mRNA encoding CD3 in UUO kidneys from WT and BLT1^-/-^ mice. Data are expressed as the mean ± SEM (WT; Day 0: n = 8, Day 1: n = 10, Day 3: n = 16, Day 5: n = 12, Day 7: n = 16 BLT1; Day 0: n = 8, Day 1: n = 12, Day 3: n = 16, Day 5: n = 11, Day7: n = 14 mice per group).

It is well known that macrophages, neutrophils and lymphocytes are related to fibrosis formation. Interstitial macrophages promote fibrosis in the UUO kidney [38]. Therefore, we asked what type of cells is most accumulated in the interstitial spaces within UUO kidneys. Immunofluorescence analysis showed that fewer F4/80-positive macrophages accumulated in interstitial tissues of BLT1^-/-^ UUO kidneys than in those of WT UUO kidneys (Fig 3A). The macrophage density in BLT1^-/-^ UUO kidneys was significantly lower than that in WT UUO kidneys from Day 3 (Day 3: 32.9±2.9 vs.24.6±3.6 cell/field, P = 0.026, Day 5: 68.9±6.8 vs.45.1±7.4, P = 0.049, Day 7: 87.2±4.1 vs.66.3±0.3, P = 0.002, WT vs. BLT1^-/-^, respectively; Fig 3B). Furthermore, expression of mRNA encoding F4/80 was significantly lower in BLT1^-/-^ UUO kidneys than in WT UUO kidneys on Days 3, 5, and 7 (Day 3: P<0.001, Day 5: P = 0.023, Day 7: P = 0.003, WT vs. BLT1^-/-^, respectively; Fig 3C).

In contrast, immunofluorescence analysis against Gr1 showed that there was no significant difference in the number of accumulated Gr1 positive cells between WT and BLTI^-/-^ mice except on Day 3 (Fig 3D and 3E). In addition, there was no significant change in the expression of mRNA encoding CD3 (Fig 3F), a specific marker of T lymphocytes. These results showed that the accumulation of macrophages in interstitial tissues was involved in fibrosis, which was dependent on LTB_4_-BLT1 signaling.

### Expression of CXCL12 in UUO kidneys is BLT1-dependent

**Fig 4:**
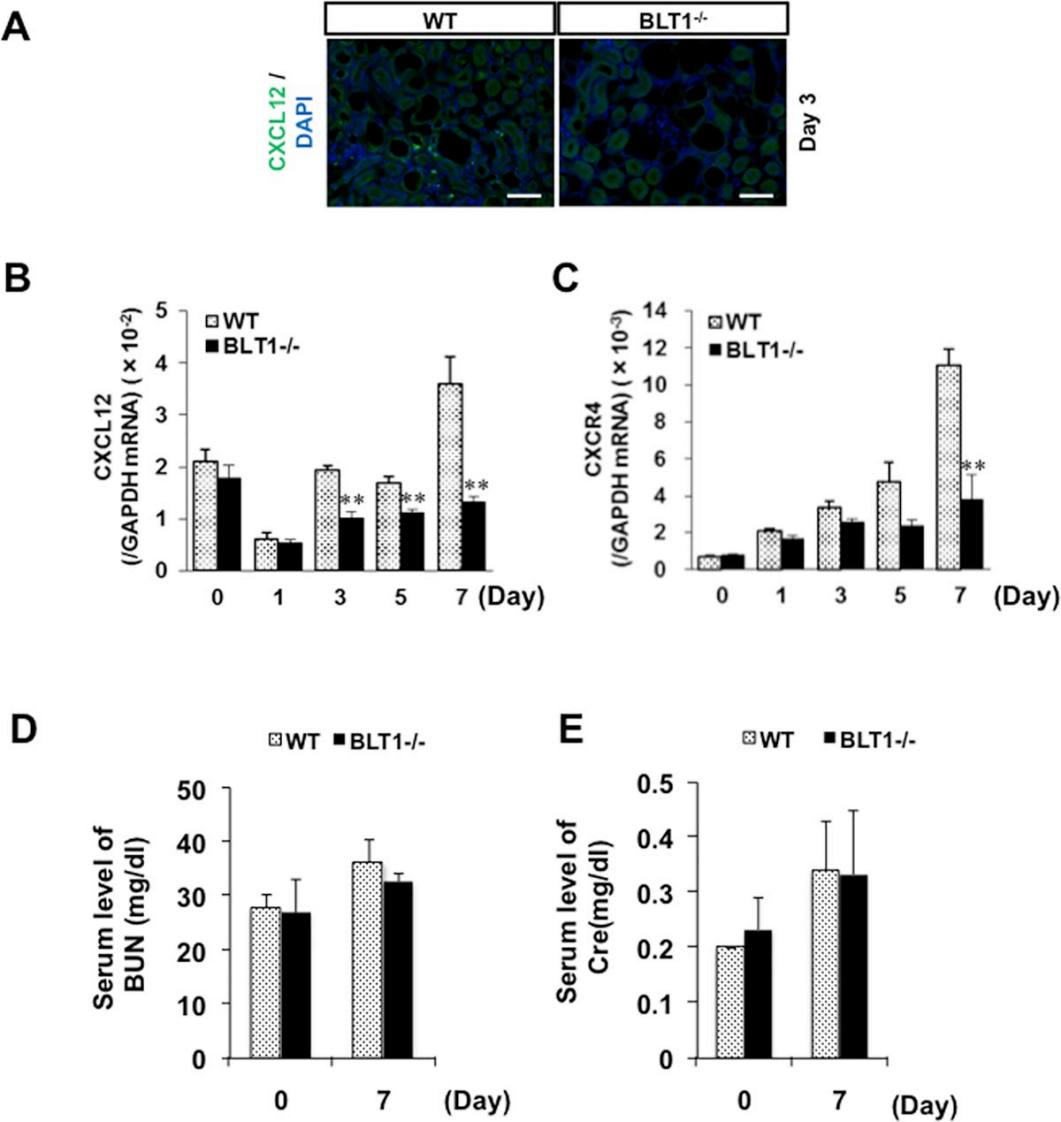
BLT1-induced CXCL12/CXCR4 axis in UUO kidneys. (A) Representative images showing immunostaining of WT UUO kidney cortex with an anti-CXCL12 antibody (A) on Day 3. CXCL12-positive cells localized to the tubulointerstitial area. Scale bars = 200 μm. (B, C) Expression of mRNA encoding CXCL12 (B) and CXCR4 (C) in UUO kidneys, as measured by real-time RT-PCR. Data are expressed as the mean ± SEM (CXCL12: WT; Day 0, 1, 3, 5, 7: n = 8 BLT1; Day 0, 1, 3, 5, 7: n = 8 mice per group. CXCR4: WT; Day 0, 1, 3, 5, 7: n = 4 BLT1; Day 0, 1, 3, 5, 7: n = 4 mice per group). **P<0.01, *vs*. WT. (D, E) Serum level of BUN (D) and Cre (E) in Day 0 and Day 7. Data are expressed as the mean ± SEM (BUN: WT; Day 0 and 7: n = 6 BLT1; Day 0 and 7: n = 4 mice per group. Cre: WT; Day 0 and 7: n = 6 BLT1; Day 0 and 7: n = 4 mice per group.).

Macrophages and fibroblasts are recruited to the kidneys following UUO [39]. Therefore, we examined chemokine levels in UUO kidneys from WT and BLT1^-/-^ mice. Immunofluorescence staining revealed that CXCL12 was expressed primarily in the interstitial spaces of WT UUO kidneys on Day 3 (Fig 4A). Expression of CXCL12 mRNA in WT mice fell transiently on Day 1, before increasing again on Day 3; however, this increase was significantly lower in BLT1^-/-^ mice (Day 3,5,7: P<0.001, WT vs. BLT1^-/-^, respectively; Fig 4B). Moreover, expression of CXCR4, a specific ligand for CXCL12, in BLT1^-/-^ UUO kidneys was significantly lower than that in WT UUO kidneys on Day 7 (P = 0.004, WT vs. BLT1^-/-^, Fig 4C). These results suggest that BLT1-induced macrophage infiltration into the UUO kidneys is dependent on the CXCL12/CXCR4 axis. There was no significant change between serum levels of BUN (Day 0: P = 0.811, Day 7: P = 0.241 Fig 4D) and Cre (Day 0: P = 0.285, Day 7: P = 0.929 Fig 4E) in WT and BLT1^-/-^ mice.

### LTB_4_ increases expression of TGF-β, FGF-2, and collagen mRNA by macrophages *in vitro*

**Fig 5:**
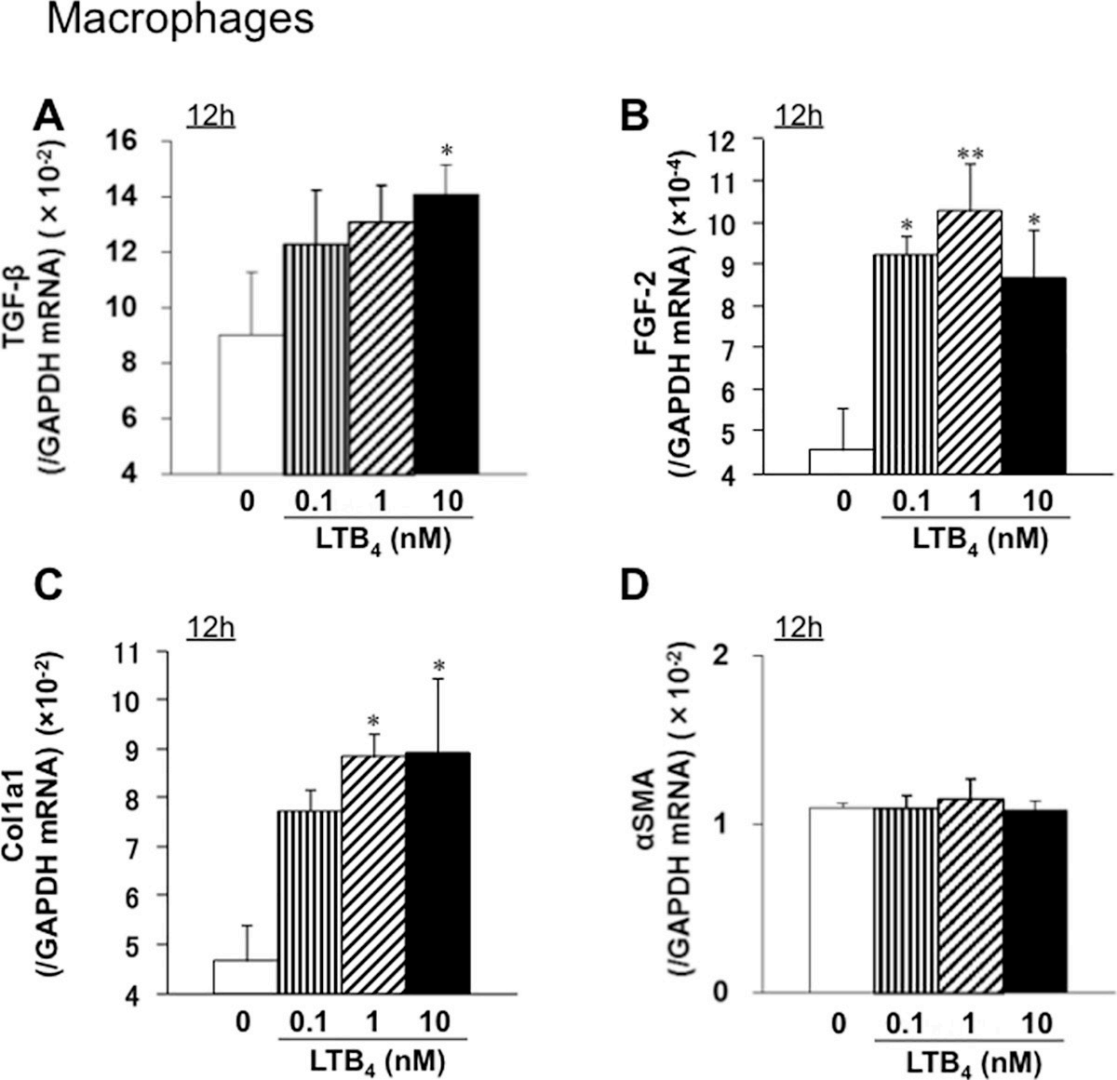
LTB_4_ increases expression of mRNA encoding TGF-β, FGF-2, and collagen by macrophages of WT mice *in vitro*. Macrophages collected from the peritoneal cavity of WT mice were incubated with LTB_4_ for 12 h. Expression of mRNA encoding TGF-β (A), FGF-2 (B), Col1a1 (C), and αSMA (D) was measured by real-time quantitative RT-PCR. Expression of mRNA encoding TGF-β, FGF-2, or Col1a1 (but not αSMA) increased after LTB_4_ treatment. Data are expressed as the mean ± SEM (n = 6). *P<0.05 and **P<0.01, *vs*. 0 nM.

To evaluate whether expression of pro-fibrotic cytokines and collagen by macrophages is dependent on LTB_4_-BLT1 signaling, we isolated macrophages of WT mice from the peritoneal cavity and incubated them with LTB_4_. Expression of mRNA encoding TGF-β and FGF-2 increased 12 h after LTB_4_ treatment (Fig 5A and 5B). Increased expression of collagen gene Col1a1 was also detected 12 h after addition of LTB_4_ (Fig 5C), although expression of αSMA did not increase after LTB_4_ treatment (Fig 5D). These results suggest that LTB_4_ induces expression of collagen, TGF-β, and FGF-2 by macrophages.

### LTB_4_ increases expression of mRNA encoding αSMA in connective tissue-derived mouse L929 fibroblast-like cells *in vitro*

**Fig 6:**
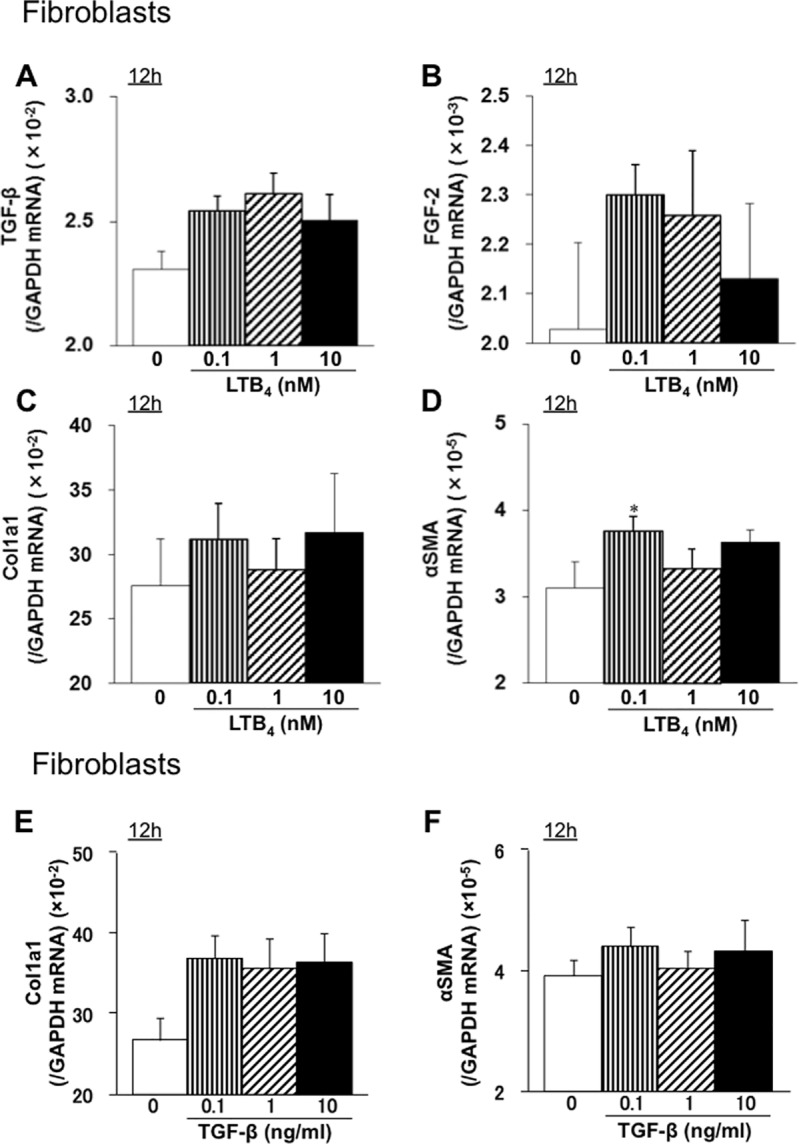
LTB_4_ increases expression of mRNA encoding αSMA in mouse L929 fibroblast-like cells derived from connective tissues. L929 mouse fibroblasts were treated with LTB_4_ for 12 h, and expression of mRNA encoding TGF-β (A), FGF-2 (B), Col1a1 (C), or αSMA (D) was measured by real-time quantitative RT-PCR. Data are expressed as the mean ± SEM (n = 6). *P<0.05, *vs*. 0 nM. L929 mouse fibroblasts were treated with TGF-β for 12 h, and expression of mRNA encoding Col1a1 (E) and αSMA (F) was measured by real-time quantitative RT-PCR. Data are expressed as the mean ± SEM (n = 6). *P<0.05, vs. 0 nM.

When L929 murine fibroblasts were incubated with LTB_4_, we observed no increase in expression of mRNA encoding TGF-β or FGF-2 (Fig 6A and 6B). Unlike in macrophages, LTB_4_ did not induce increased expression of mRNA encoding collagen 1a1, although αSMA mRNA levels increased slightly at 12 h after addition of 0.1 nM LTB_4_ (Fig 6C and 6D). These results suggest that LTB_4_ does not trigger secretion of collagen 1, TGF-β, or FGF-2 by L929 cells. When L929 cells were stimulated with TGF-β, we saw no change in expression of mRNA encoding Col1a1 and αSMA (Fig 6E and 6F).

### Accumulation of BM-derived macrophages induced by LTB_4_-BLT1 signaling exacerbate fibrosis in the UUO kidneys

**Fig 7:**
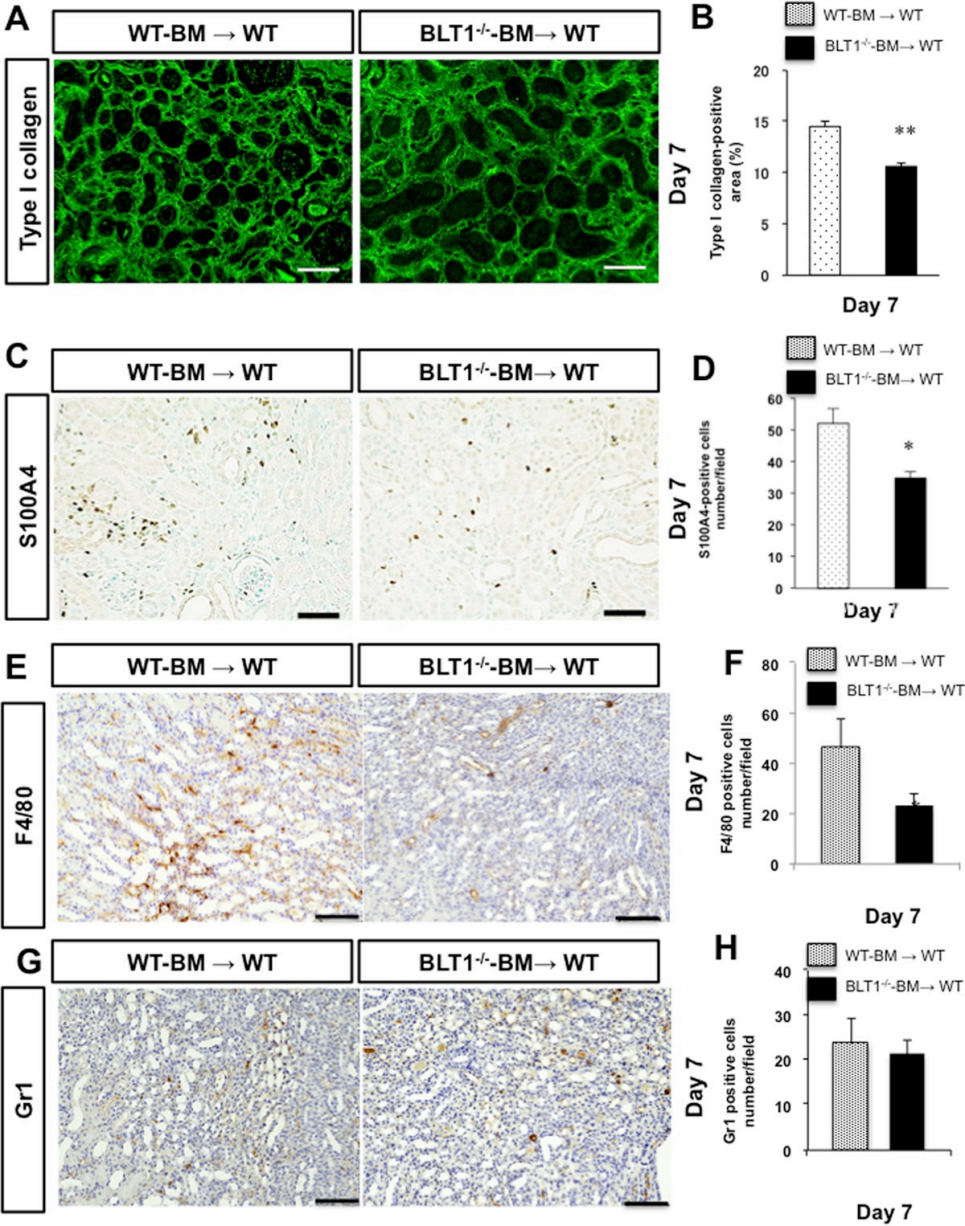
Bone marrow-derived cells induced by LTB_4_-BLT1 signaling exacerbate renal fibrosis in UUO kidneys. We examined renal fibrosis in UUO kidneys of WT mice transplanted with BLT1^-/—^BM (BLT1^-/—^BM→WT), and compared it with that in WT mice transplanted WT-BM (WT-BM→WT). (A) Representative images showing type I collagen staining in the UUO kidneys of WT-BM→WT (left panel) and BLT1^-/—^BM→WT (right panel) at Day 7. Scale bars = 200 μm. (B) The type I collagen-positive area in UUO kidneys from WT-BM→WT and BLT1^-/—^BM→WT at Day 7. Data are expressed as the mean ± SEM (n = 4 mice/group). **P<0.01, *vs*. WT-BM→WT mice. (C) Representative images showing S100A4 immunostaining in UUO kidneys of WT-BM→WT and BLT1^-/—^BM→WT at Day 7. Scale bars = 200 μm. (D) Number of S100A4-positive cells in UUO kidneys from WT-BM→WT and BLT1^-/—^BM→WT. Data are expressed as the mean ± SEM (n = 4 mice/group). **P<0.01, *vs*. WT-BM→WT. (E) Representative images showing F4/80 immunostaining in UUO kidneys of WT-BM→WT and BLT1^-/—^BM→WT at Day 7. Scale bars = 100 μm. (F) Number of F/80-positive cells in UUO kidneys of WT-BM→WT and BLT1^-/—^BM→WT. Data are expressed as the mean ± SEM (n = 4 mice/group). **P<0.01, *vs*. WT-BM→WT. (G) Representative images showing Gr1 immunostaining in UUO kidneys of WT-BM→WT and BLT1^-/—^BM→WT at Day 7. Scale bars = 100 μm. (H) Number of Gr1-positive cells in UUO kidneys of WT-BM→WT and BLT1^-/—^BM→WT. Data are expressed as the mean ± SEM (n = 4 mice/group). **P<0.01, *vs*. WT-BM→WT.

Next, we examined whether BM cells induced by LTB_4_-BLT1 affect renal fibrosis. Selective deletion of the BLT1 receptor from the BM was performed by transplantation of BM cells from BLT1^-/-^ mice. BM transplantation revealed that the area of type I collagen deposition in BLT1^-/—^BM→WT on Day 7 was significantly smaller than that in WT-BM→WT (14.5±0.5 vs.10.6±0.3%, P = 0.0006, WT-BM→WT vs. BLT1^-/—^BM→WT, Fig 7A and 7B). In addition, accumulation of S100A4-positive cells in BLT1^-/—^BM→WT at Day 7 was significantly lower than that in WT-BM→WT (52±4.7 vs.34.8±2.0 cells/field, P = 0.015, WT-BM→WT vs. BLT1^-/—^BM→WT, Fig 7C and 7D). Next, we confirmed whether BM-derived macrophages accumulated in UUO kidneys or not. Accumulation of F4/80 positive cells in BLT1^-/—^BM→WT at Day 7 was significantly lower than that in WT-BM→WT (46±11.4 vs.23.2±4.7 cells/field, P = 0.0004, WT-BM→WT vs. BLT1^-/—^BM→WT, Fig 7E and 7F). There is no significant change in accumulation of Gr1 positive cells between BLT1^-/—^BM→WT and WT-BM→WT (23.7±5.6 vs.21.3±3.2 cells/field, P = 0.361, WT-BM→WT vs. BLT1^-/—^BM→WT, Fig 7G and 7H). These results suggested that BM-derived F4/80 positive macrophages expressing BLT1 contributed to development of renal fibrosis in the UUO kidney.

## Discussion

**Fig 8:**
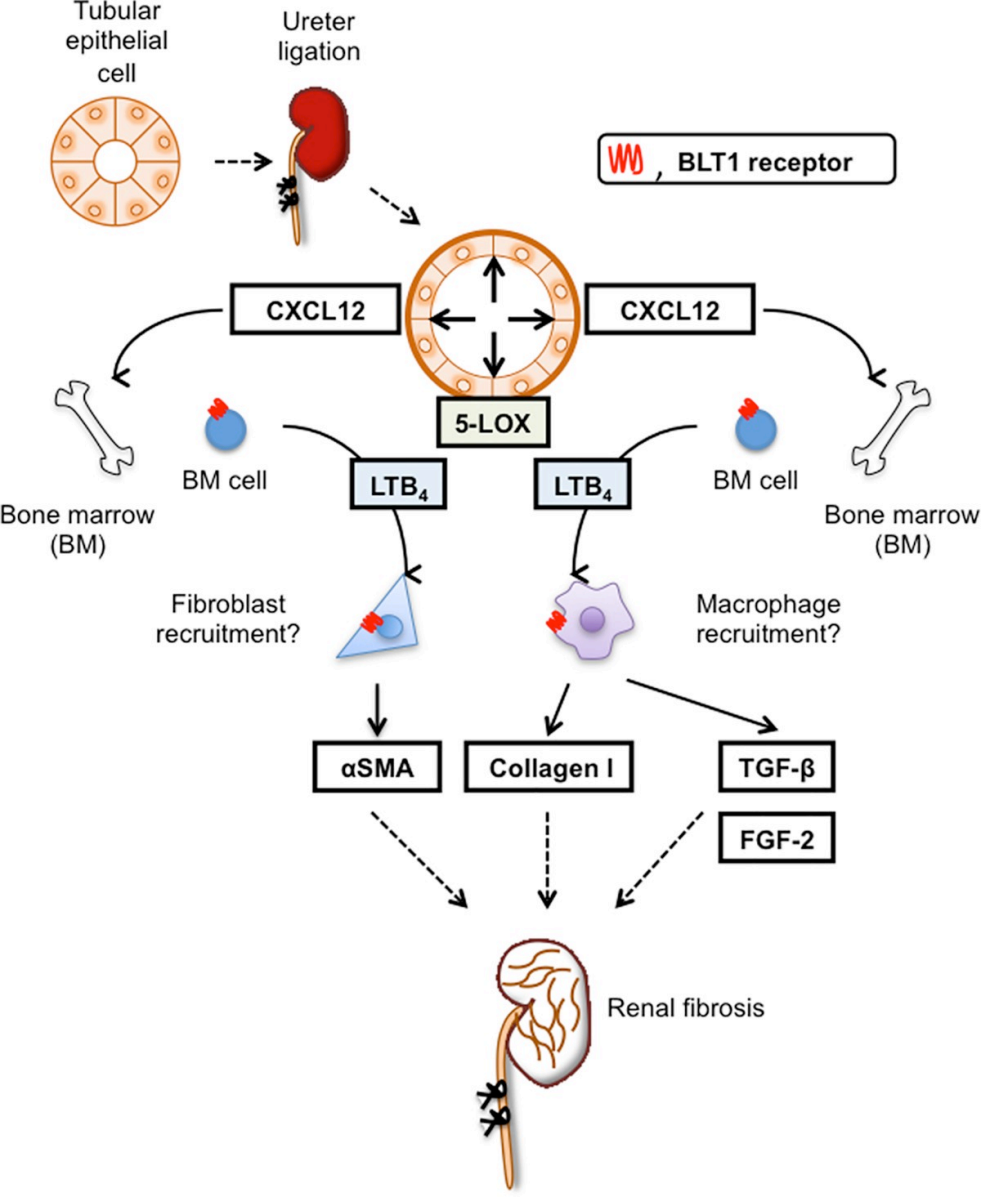
Role of LTB_4_-BLT1 signaling in UUO kidney fibrosis. BLT1 signaling is critical for development of fibrosis after UUO treatment. UUO treatment upregulates expression of CXCL12 in a BLT1-specific manner. LTB_4_ is also induced by UUO treatment. LTB_4_-BLT1 signaling increases recruitment of fibrocytes and macrophages to the kidney; these cells produce collagen, together with TGF-β and FGF-2, via BLT1 signaling, resulting in fibrotic changes.

Here, we demonstrated that BLT1 signaling plays a major role in development of fibrosis in BLT1^-/-^ UUO model mice. Accumulation of collagen type I in UUO kidneys was significantly lower in BLT1^-/-^ mice than in WT mice. We also observed BLT1-dependent recruitment of macrophages and fibroblasts (Fig 2 and Fig 3); however, BLT1 signaling induced expression of mRNA encoding pro-fibrotic factors and collagen in a cell type-specific manner (Fig 2). Surprisingly, we found that LTB_4_ acted on macrophages directly to upregulate expression of Col1a1. BLT1 signaling also induced expression of mRNA encoding TGF-β and FGF-2 *in vivo*, both of which are pro-fibrotic factors; however, we did not demonstrate production of TGF-β and FGF-2 by LTB_4_-stimulated fibroblasts *in vitro* (Fig 5 and Fig 6). BM transplantation experiments revealed that the area of type I collagen deposition in UUO kidneys from BLT1^-/—^BM→WT was lower than that from WT-BM→WT (Fig 7). Thus, BM-derived cells induced by LTB_4_-BLT1 signals exacerbate renal fibrosis in the UUO kidney. Together, these results demonstrated a finely tuned mechanism underlying BLT1-dependent fibrosis in this model (Fig 8), and suggested that blocking of BLT1 signaling may prevent fibrosis.

The UUO model is good for studying tubulointerstitial fibrosis accompanied by cellular infiltration. LTB_4_ is a chemoattractant for leukocytes, particularly neutrophils and macrophages [21–24]. We found that, after UUO treatment, dilated tubular cells expressed 5-LOX (Fig 1G), which may be important for initiating BLT1-dependent fibrosis. Expression of 5-LOX also induces LTB_4_, a chemoattractant for macrophages. These molecules elicit local extravasation of fibroblasts and macrophages, which infiltrate the tubulointerstitial space of the kidney in a BLT1-dependent manner.

PGE_2_ and PGI_2_, the most abundant eicosanoids, suppress fibrosis *in vivo* [18, 40]; however, few studies have examined involvement of LTs and other metabolites in fibrosis after UUO. Models of pulmonary fibrosis exhibit a synthetic imbalance favoring pro-fibrotic LTs over anti-fibrotic PGE_2,_ suggesting a role for eicosanoids in fibrotic lung disease [41]. PGE_2_ levels in BAL fluid from patients with idiopathic pulmonary fibrosis (IPF) are lower than those from healthy control subjects [42]. In addition, fibroblasts grown from lung tissue isolated from patients with IPF synthesize less PGE_2_ than cells from healthy control subjects due to reduced COX-2 expression [43]. This has important pathologic consequences because decreased levels of COX-2 and PGE_2_ in these cells contribute to increased collagen synthesis and cell proliferation in response to TGF-β [43]. By contrast, BAL fluid from patients with IPF contains more LTB_4_ than that from control subjects [44]. LTB_4_ levels in lung tissue homogenates from patients with IPF are 15-fold higher, and those of LTC_4_ are 5-fold higher, than those in control subjects, reflecting constitutive activation of 5-LOX in alveolar macrophages [45]. Increased lung LT levels have been observed in mice after intratracheal administration of bleomycin; this is a commonly used animal model of pulmonary fibrosis [46]. In this model, fibrosis was blunted markedly following disruption of cPLA_2_ [47] and 5-LOX [46], suggesting that endogenous LTs play a major role in facilitating fibrosis. Several features of bleomycin-induced injury, including pulmonary recruitment of macrophages and neutrophils, alveolar septal thickening, fibroblast accumulation, and collagen deposition, are significantly less severe in LT receptor-deficient mice than in their WT littermates [48]. These results suggest that LTs are pro-fibrotic in these pathological settings. In contrast to lung fibrosis, there is no definitive evidence that LT is involved in UUO-induced fibrosis in the kidneys.

As mentioned above, we showed that 5-LOX expression increased after induction of UUO; we also observed 5-LOX-expressing cells in dilated renal tubules in UUO kidneys (Fig 1G). A previous study in a mouse UUO model shows that COX-2 is upregulated in renal tubule epithelial cells after ligation of the ureter [18].

We observed BLT1-dependent accumulation of type I collagen, S100A4-positive fibroblasts, and αSMA-positive myofibroblasts in UUO kidneys (Fig 2). Accumulation of S100A4-positive fibroblasts in the interstitial spaces within kidney tissues was evident from the early stages of UUO onset, although it was less marked in BLT1^-/-^ mice. The αSMA mRNA levels (Fig 2H) suggest that αSMA-positive myofibroblasts accumulate in UUO kidneys; again, this was less marked in BLT1^-/-^ mice from Day 3. These results suggest that LTB_4_/BLT1 signaling is important for induction of renal fibrosis in UUO kidneys.

We also observed reduced accumulation of macrophages in BLT1^-/-^ mice during the early stage of UUO (Fig 3). In contrast, there was no difference in the number of accumulated Gr1-positive cells between WT mice and BLT1^-/-^ mice except on Day 3. Although neutrophils are the first responder cell type to be recruited to the inflammatory sites contributing to tissue injury in a BLT1 dependent manner [49], our data demonstrated that infiltration by macrophages in UUO kidneys appeared to be extensive as compared with neutrophils during the development of fibrosis formation. These results suggested that the accumulated macrophages mainly are involved in fibrosis formation. These accumulated macrophages supply TGF-β to sites of fibrosis. CXCL12 is a chemokine that plays a role in migration of BM-derived stem cells to the peripheral blood and from there to sites of tissue injury. CXCL12 is a potent chemoattractant for fibroblasts/myofibroblasts. CXCL12 binds to a specific receptor, CXCR4. Philips et al. report that the CXCL12/CXCR4 axis induces recruitment of BM-derived stem cells to injured lung tissue to induce pulmonary fibrosis [50]. Also, CXCR4 antagonists ameliorate renal fibrosis in the UUO kidney [51]. It would be worth investigating whether LTB_4_-BLT1 signaling interacts with the CXCL12/CXCR4 axis in the UUO kidney. Here, we found that CXCL12-positive cells localized primarily to the interstitial spaces within UUO kidneys (Fig 4A). Furthermore, expression of CXCL12 and CXCR4 increased in WT kidneys more than in BLT1^-/-^ kidneys. These results suggested that the CXCL12/CXCR4 axis contributes to renal fibrosis in the UUO kidney in a LTB_4_-BLT1 signaling-dependent manner. The results also suggested that accumulation of collagen-producing cells is regulated by BLT1 signaling. Lack of BLT1 signaling may explain, at least in part, the reduced fibrosis observed in UUO kidneys. BM-mobilized macrophages and fibrotic cells express CXCR4 and infiltrated CXCL12-enriched tissue; however, we did not identify the type of cell that contributes to renal fibrosis in UUO kidney. Further experiments are needed to answer this question.

TGF-β has the potential to increase collagen biosynthesis by fibroblasts and myofibroblasts [52]. We confirmed reduced expression of TGF-β in UUO kidneys of BLT1^-/-^ mice, along with increased expression of TGF-β by macrophages stimulated with LTB_4_ *in vitro* (Fig 5A). Interestingly, LTB_4_-stimulated L929 cells did not show increased expression of TGF-β and FGF-2 (Fig 6A and 6B). Moreover, LTB_4_-stimulated macrophages upregulated expression of collagen 1a mRNA, but not that of αSMA mRNA (Fig 5C and 5D). By contrast, LTB_4_-stimulated L929 cells upregulated expression of αSMA mRNA, but not collagen 1a mRNA (Fig 6C and 6D). These results suggest that LTB_4_-BLT1 signaling induces fibrosis via accumulation of macrophages and fibroblasts. A previous report suggested that LTB_4_ did not alter expression of collagen-encoding genes in primary mouse lung fibroblasts. Furthermore, bleomycin-treated macrophages produced LTB_4_ and showed increased production of TGF-β in a BLT1-dependent manner [53]. Taken together, these results suggest that LTB_4_-BLT1 signaling might promote TGF-β production by macrophages recruited via BLT1 signaling.

The results of the BM transplant experiments suggest that BM-derived cells induced by LTB_4_-BLT1 signaling exacerbate renal fibrosis in the UUO kidney (Fig 7). Immunohistochemical study showed that accumulation of F4/80-positive macrophages in UUO kidneys in BLT1^-/—^BM→WT at Day 7 was significantly lower than that in WT-BM→WT (Fig 7E and 7F). Accumulation of BM-derived macrophages expressing BLT1 was associated with collagen deposition in the UUO kidney. These results indicated that BLT1 signaling in BM-derived macrophages is involved in renal fibrosis. In addition, the current study showed that there was no change in the number of Gr1 positive neutrophils in the UUO kidneys between BLT1^-/—^BM→WT and WT-BM→WT on Day 7 (Fig 7G and 7H). In this experiment, we did not estimate accumulation of neutrophils in the early phase. Further study is needed to answer whether Gr1 positive neutrophils related to fibrosis formation in the UUO kidney. Taken together, the results from the present experiments suggest that complex synergistic loops may be active during LTB_4_-induced fibrosis in this UUO model.

In conclusion, we showed here that BLT1 signaling plays a role in development of fibrosis in UUO models. Accumulation of collagen type I in UUO kidneys of BLT1^-/-^ mice was significantly lower than that in UUO kidneys of WT mice. BLT1 signaling induced accumulation of macrophages and fibroblasts, and induced collagen biosynthesis directly via induction of TGF-β. Thus, BLT1-dependent fibrosis in this model is finely regulated, suggesting that BLT1 signaling is a good therapeutic target for preventing fibrosis.

## Abbreviations

BLT1: the high-affinity leukotriene B_4_ receptor
BLT1^-/-^: BLT1 knockout mice
UUO: unilateral ureteral obstruction
5-LOX: 5-lipoxygenase
CXCL12: C-X-C motif chemokine 12
CXCR4: C-X-C chemokine receptor type 4
TGF-β: transforming growth factor-β
FGF-2: fibroblast growth factor 2
