# Correction: Role of the high-affinity leukotriene B_4_ receptor signaling in fibrosis after unilateral ureteral obstruction in mice

**DOI:** 10.1371/journal.pone.0215625

**Published:** 2019-04-15

**Authors:** Mariko Kamata, Hideki Amano, Yoshiya Ito, Tomoe Fujita, Fumisato Otaka, Kanako Hosono, Kouju Kamata, Yasuo Takeuchi, Takehiko Yokomizo, Takao Shimizu, Masataka Majima

The Funding statement is incorrect. The correct Funding statement is as follows: This study was supported by Grants-in-Aid for Scientific Research, grant numbers: 17K16093 to Dr. Mariko Kamata; 15H05904, 18H02627 to Dr. Takehiko Yokomizo; 23116102 to Dr. Masataka Majima; 26462132 to Dr. Hideki Amano; and 26293055, 18H02605 to Dr. Masataka Majima.
